# Supplementary material for: Osmunda japonica Extract Suppresses Pro-Inflammatory Cytokines by Downregulating NF-κB Activation in Periodontal Ligament Fibroblasts Infected with Oral Pathogenic Bacteria
Source: Int J Mol Sci. 2020 Apr 1;21(7):2453. doi: 10.3390/ijms21072453 (PMC7177349; doi:10.3390/ijms21072453)
Supplement: Supplementary file 1 [file ijms-21-02453-s001.pdf]

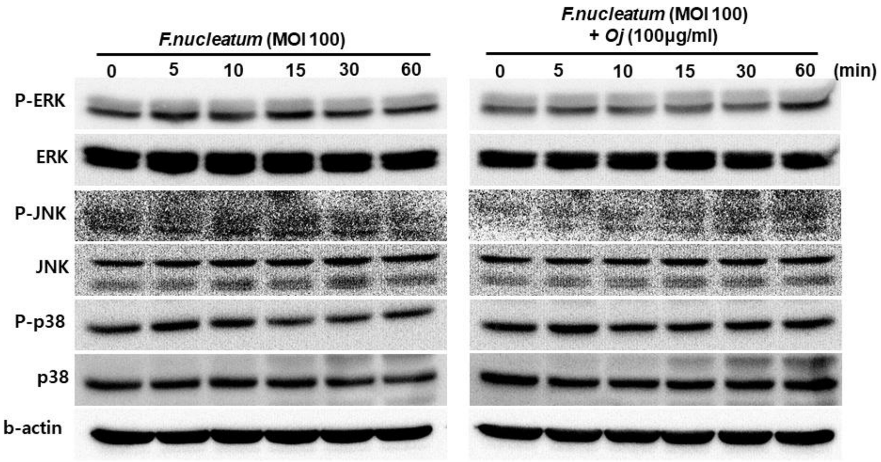

Supplementary Figure 1. The effect of *OJE* on phosphorylation of MAPKs. Western blotting was performed to detect the activation of ERK, JNK, and p38 in response to *F.nucleatum infection* under the absence or presence of OJE. Human PDLFs were pre-treated with 100 µg/mL *OJE* prior to bacterial infection (M.O.I=100). *O. japonica*: Oj; *F. nucleatum*: Fn
